# Diagnostic yield and appropriate indication of upper endoscopy in Jordanian children

**DOI:** 10.1186/s12887-020-02470-6

**Published:** 2021-01-05

**Authors:** Eyad Altamimi, Yousef Odeh, Tuka Al-quraan, Elmi Mohamed, Naif Rawabdeh

**Affiliations:** 1 Pediatric Department, Faculty of Medicine, Jordan University of Science and Technology, P.O.Box. 3030 , Postal code: 22110 Irbid, Jordan; 2 Pediatric Department, King Abdullah University Hospital, Ar Ramtha, Jordan

**Keywords:** Endoscopy, Appropriate, Children, Histology, Gastritis, Abdominal pain, Children

## Abstract

**Background:**

Upper endoscopy is an essential tool for diagnosing pediatric gastrointestinal issues. This study aimed to assess the indications, diagnostic yields, concordance between histopathological and endoscopic findings and suitability of upper endoscopies performed at a tertiary university hospital in Jordan.

**Methods:**

Hospital records of children who underwent upper endoscopy were retrospectively reviewed. Demographics, endoscopic details (e.g., indications, findings and any complications), and histopathological findings were collected. The relationship between endoscopic findings and histopathological abnormalities was reported.

**Results:**

The study included 778 patients (age, 92.5 ± 54.5 months; 380 girls, 48.8%). The most common age group was children younger than 60 months (273 patients, 34.3%). The most common indication for endoscopy was abdominal pain, followed by vomiting and failure to thrive or weight loss. Normal upper endoscopy was reported in 411 patients (52.8%). Age below 60 months, abdominal pain, dysphagia/odynophagia, and heartburn were predictive of abnormal endoscopy in multivariate analysis with p-value 0.000, 0.048, 0.001 and 0.01 respectively. Abnormal endoscopy showed 67.3% sensitivity and 69.9% specificity to predict histopathological abnormalities. Of those performed, 13.6% endoscopies were described as inappropriate indication. The suitability of the procedure was a sensitive predictor for abnormal endoscopic and histopathological findings.

**Conclusions:**

Abdominal pain is the most common indication for upper endoscopy in our population. It is associated with a higher chance of abnormal endoscopy. Concordance between endoscopic and histopathological findings is not high. Normal endoscopic findings shouldn`t discourage the endoscopist from obtaining tissue biopsies. Considering more biopsies may improve pathological detection rates. Compliance with established endoscopy guidelines may reduce unnecessary procedures.

## Background

With the widespread availability of upper endoscopy, an increasing number of children are referred for endoscopic evaluation. Upper endoscopy allows for the direct examination and obtaining tissue biopsies. [1] Coupling endoscopy with histological examination increases the yield and precision of the diagnosis. In low-resource countries with financial hardships, this expansion places significant pressure on parents, the endoscopy team, and the health sector.

The European Society of Gastrointestinal Endoscopy (ESGE) and European Society for Pediatric Gastroenterology, Hepatology, and Nutrition (ESPGHAN) have provided evidence-based guidelines with clear indications and timing of diagnostic and therapeutic endoscopy in pediatric patients. [2] Multiple studies have examined the reliability of endoscopic indications in children against published guidelines. Lee et al. reported a high rate of compliance (99.7%) with published guidelines in their cohort. This compliance predicted positive endoscopic findings, but didn`t change the initial clinical diagnosis or therapeutic plan very much. [3] Jantchou et al. found that compliance with published guidelines is associated with better diagnostic efficiency.[4] On the other hand, Miele et al. examined the effect of publishing ROME criteria on endoscopic practice, they found reduction in the inappropriate procedures since publishing the criteria, on the other hand; appropriate procedures were more contributive compared to inappropriate ones. [5]

This study focused on reviewing our pediatric patients who underwent upper endoscopy in King Abdullah University Hospital (KAUH) during the study period, and it aimed at describing the patients’ characteristics, endoscopic and histopathological findings, the discrepancy between normal endoscopic finding and abnormalities in histology as well as evaluating the suitability of the procedures performed.

## Methods

This is a retrospective, chart-based study conducted in the Pediatric Gastroenterology Division of the Pediatrics Department at Jordan University of Science and Technology (JUST) and King Abdullah University Hospital (KAUH) (Ar Ramtha, Jordan). The study was approved by the institutional review board of the Faculty of Medicine and the Research Committee at JUST (approval no. 20,190,077).

All procedures were completed by senior pediatric gastroenterologists (EA, NR) in the pediatric endoscopy unit at KAUH. Patients underwent the procedures under light sedation with intravenous midazolam and local lidocaine spray. Heavier sedation (ketamine/fentanyl) was used on a case-specific basis.

Histopathological assessments were performed in the histopathology department at KAUH by a senior pathologist, pediatric pathologist, or gastrointestinal pathologist.

For this study, all patients who underwent upper endoscopy by a pediatric gastroenterologist at KAUH between January 2014 and January 2020 were identified. Electronic files, including clinical/inpatient files, endoscopic reports, and histopathological reports, were reviewed. For each patient, the following data were retrieved: age, sex, presenting symptoms, examination findings, laboratory test results, endoscopy abnormalities, histopathology findings, and adverse events (sedation or procedure related). The endoscopy procedure list contains both patients evaluated by the pediatric gastroenterologists and referrals.

Therapeutic endoscopy cases (retrieval of a foreign body, variceal ligation, stricture dilatation) were excluded. For patients who underwent multiple endoscopic procedures (whether as a follow-up or due to the persistence of the complaint), only the first diagnostic procedure was included. Patients who underwent endoscopy outside our facility were not considered. In addition, patients who underwent prior surgical procedures leading to expected abnormal endoscopy (for example; gastrectomy) were excluded. Procedures with insufficient data (endoscopy report or pathological report) were excluded.

We opted to use the endoscopy reports for comparison because not all patients undergoing endoscopy procedures had records of a histopathological examination.

### Definitions

An endoscopy was categorized as normal when the upper endoscopy report mentioned either no abnormality or only a minor abnormality that did not contribute to the patient’s illness. It was categorized as abnormal when the upper endoscopy report mentioned an abnormality in any of the areas examined. Histopathology was classified as negative when a histopathological report described no abnormalities or only a mild nonspecific abnormality and positive when the report described abnormal findings.

Indication for the procedure was described as appropriate when compliant with the 2016 Pediatric Gastroenterology Endoscopy Guidelines by the ESGE and ESPGHAN, whereas inappropriate indication described noncompliance.[2].

### Statistical Analysis:

Data were analyzed using IBM SPSS Statistics20.0 software (IBM Co., Armonk, NY, USA). Descriptive data were reported as percentages of the total for categorical data, as means ± standard deviation (SD). T-test, x^2^ and fisher exact test were used as appropriate to compare categories, p-value of < 0.05 considered significant. Variables identified to be significant in the univariate analysis were used as predictors of the probability of abnormal EGD findings or abnormal histopathology in the multivariate analysis.

## Results

During the study period, 1,072 upper endoscopy procedures (as some patients had more than one procedures) were identified. After 313 were excluded according to the predetermined criteria (212 therapeutic/potentially therapeutic endoscopy, 23 previous upper gastrointestinal surgeries and 40 due to insufficient data), 787 patients were included in the study (average age ± SD, 92.5 ± 54.5 months; 380 girls, 48.8%). As shown in Table 1, the most common age group was children younger than 60 months (267 patients, 34.23%).

**Table 1:** Demographics of pediatric patients who underwent upper endoscopy from January 2014 to January 2020 (*N* = 778)

*Age*				
Mean ± SD (range)	92.5 ± 54.5 months (2 days–216 months) (2days-18 years)			
*Age distribution*				
< 60 months (n, %)	(267, 34.3%)			
60–120 months (n, %)	(230, 29.6%)			
120–180 months (n, %)	(264, 33.9%)			
≥ 180 months (n, %)	(17, 2.2%)			
*Sex*				
Female	380 (48.8%)			
Male	398 (51.2%)			
*Primary indication for upper endoscopy*^a^		*N*		*%*
Abdominal pain/dyspepsia		359		45.1
Vomiting		164		21.1
Failure to thrive/weight loss		80		10.3
Chronic diarrhea		66		8.5
R/O celiac disease		53		6.8
Anemia		49		6.3
Abdominal distention		37		4.8
Dysphagia/odynophagia		42		5.4
Chemical ingestion		31		4.0
Chronic constipation		10		1.3
Heartburn		11		1.4
Allergies		6		0.8
Poor appetite		5		0.6
Other		21		2.9

^a^Some patients may have more than one indication

The most common indication for upper endoscopy in our cohort was abdominal pain (*n* = 359; 45.1%), followed by vomiting (*n* = 164; 21.1%) and failure to thrive or weight loss (*n* = 80; 910.3%) (Table 1).

Normal upper endoscopy was reported in 411 (52.8%) patients. Children with normal endoscopy were significantly younger (*p* = 0.001). Neither sex nor the number of identified indications for endoscopy showed any statistical difference between the normal and abnormal upper endoscopy groups. (Table 2).

**Table 2:** Comparison between patients with normal and abnormal endoscopy findings regarding patients` characteristics and procedures` indication(s)

Feature	Normal endoscopy findings (*n* = 411) 52.8%	Abnormal endoscopy findings (*n* = 367) 47.2%	*p*-value
Age (average ± SD)	78.1 ± 51.6	107.7 ± 53.6	0.000
<60 months †	177 (42.4%)	90 (23.7%)	0.000
60–120	133 (31.9%)	104 (27.4%)	0.165
120–180	92 (22.1%)	158 (41.6%)	0.000
≥180	15 (3.6%)	16 (4.2%)	0.662
Female	206 (49.4%)	183 (47.4%)	0.554
Single indication	331 (79.4%)	289 (76.1%)	0.263
Two indications	76 (18.2%)	80 (21.1%)	0.303
Three indications	10 (2.4%)	11 (2.9%)	0.660
Abnormal histopathology^a^	158 (28.3%)	309 (49.7%)	0.001
Abdominal pain/dyspepsia †	158 (38.6)	210 (55.3)	0.000
Vomiting	85 (20.4)	74 (19.5)	0.715
Failure to thrive/weight loss	51 (12.2)	24 (6.3)	0.005
Chronic diarrhea	42 (10.1)	19 (5.0)	0.007
R/O celiac disease	33 (7.9)	19 (5.0)	0.098
Anemia	27 (6.5)	22 (5.8)	0.682
Abdominal distention	25 (6.0)	12 (3.2)	0.062
Dysphagia/odynophagia†	11 (2.6)	30 (7.9)	0.001
Chemical ingestion	15 (3.6)	15 (3.9)	0.827
Chronic constipation	4 (1.0)	6 (1.6)	0.453
Heartburn †	1 (0.2)	10 (2.6)	0.003
Allergies	6 (1.4)	1 (0.3)	0.225
Poor appetite	3 (0.7)	1 (0.3)	0.428
Other	10 (2.4)	11 (2.9)	0.661

^a^Some patients may have more than one biopsy

†Predictive of abnormal endoscopy in multivariate analysis (*p* vale < 0.02)

Comparing the endoscopic indications between the normal endoscopy and abnormal endoscopy groups, failure to thrive or weight loss and chronic diarrhea were significantly associated with normal endoscopy (*p* = 0.005 and 0.007, respectively). By contrast, abdominal pain, dysphagia/odynophagia, and heartburn were significantly associated with abnormal endoscopy (*p* = 0.001, 0.001, and 0.003, respectively) (Table 2).

Abnormal histological findings were seen in 467 (39.6%) of the examined tissue biopsies. The stomach was the most common site of reported endoscopic abnormalities (267, 32.4%) and the highest yielding site of histopathological biopsies (267/421, 63.4%), followed by the esophageal and duodenal sites, respectively (Table 3). Abdominal pain, vomiting, dysphagia, and heartburn were associated with abnormal histological findings, (*p* = 0.0001, 0.001, 0.044 and 0.020 respectively (Table 4). Abnormal endoscopic findings were associated with higher rates of abnormal histology with a positive predictive value of 76.8%.tive (Table 5).

**Table 3:** Comparison of endoscopic and histopathological findings by organ

	Endoscopic findings	Rate^a^	Histopathological findings	Rate^a^
Esophagus	- Erythema - Ulcers - Strictures - Exudates - Furrows, whitish plaques - Circular rings	117/787 (15%)	- Reflux esophagitis - Eosinophilic esophagitis - Infectious esophagitis - Nonspecific inflammation	100/214 (46.7%)
Stomach	- Hyperemia - Ulcers - Nodularity - Erosions - Hemorrhagic spots - Abnormal folds - Trichobezoar	258/787 (33.2%)	- Mild–moderate nonspecific inflammation - *H. pylori*–related gastritis - Menetrier disease - Eosinophilic gastropathy	267/421 (63.4%)
Duodenum	- Hyperemia - Erosions - Ulcers - Scalloping and ridging - Snowflake appearance - Flattened villi	115/787 (14.8%)	- Moderate to severe inflammation - Villous atrophy - Giardia - Lymphangiectasia	100/545 (18.3%)

^a^Rate of abnormal finding out of total reports (endoscopic or histopathologic if present)

**Table 4:** Comparison of normal and abnormal histological findings by indication

Indication (n)	Normal histopathological findings	Abnormal histopathological findings	*p*-value
Abdominal pain/ dyspepsia (347)	108 (31.1%)	239 (68.9%)	0.0001
Vomiting (118)	36 (30.5%)	82 (69.5%)	0.001
Failure to thrive/weight loss (75)	35 (46.7%)	40 (53.3%)	0.569
Chronic diarrhea (60)	36 (60%)	24 (40%)	0.119
R/O celiac disease (51)	24 (47%)	27 (53%)	0.669
Anemia (49)	19 (38.8%)	30 (61.2%)	0.114
Abdominal distention (37)	19 (51.4%)	18 (48.6%)	0.856
Dysphagia/odynophagia (31)	10(32.2%)	21(67.8%)	0.044
Chemical ingestion (1)	1(100%)	0	---
Chronic constipation (8)	4(50%)	4(50%)	1.00
Heartburn (11)	2(18.2%)	9(81.8)	0.020
Allergies (6)	4(66.7%)	2(33.3%	0.416
Poor appetite (4)	3(75%)	1(25%)	0.332

**Table 5:** Diagnostic yield of endoscopic procedures^a^

	Abnormal histopathology (n)	Normal histopathology (n)
Abnormal endoscopy	272	83
Normal endoscopy	133	190
Sensitivity (%, 95%CI)	67.3% (62.43–71.81%)	
Specificity(%, 95% CI)	69.9% (64.02–75.25%)	
Positive Predictive Value %	76.8%	
Negative predictive value%	59.0% (55.08–62.82%)	

^a^Biopsies were not taken in 122 procedures

Of the 410 who had normal endoscopic findings, abnormal histological findings were reported in 133 (32.2%). This has resulted in a change in therapeutic plan in 56 cases. H.pylori gastritis was the most common finding followed by celiac disease and reflux esophagitis. (Table 6)

**Table 6:** Characteristics of patients with abnormal histopathological examination while normal endoscopy (*N* = 133)

*Age*				
Mean ± SD (range)	89.25 ± 50.82 months (5 − 192 months) (5 mo. -16 yrs)			
*Age distribution*				
< 60 months (n, %) *	(43, 32.6%)			
60–120 months (n, %)	(50, 37.6%)			
120–180 months (n, %)	(36, 27.3%)			
≥ 180 months (n, %)	(4, 3.0%)			
*Sex*				
Female	64 (48.5%)			
Male	68 (51.5%)			
*Primary indication for upper endoscopy†*		*N*		*%*
Abdominal pain/dyspepsia *		68		34.8
Vomiting		30		22.7
Failure to thrive/weight loss		11		8.3
Chronic diarrhea		10		7.6
R/O celiac disease		14		10.6
Anemia		9		6.8
Abdominal distention		6		4.5
Dysphagia/odynophagia		3		2.3
Short stature		4		3.0
Recurrent ulcers		3		2.3
Others		5		3.8
Site of Histopathological abnormalities				
Esophagus		25		
Stomach		71		
Duodenum		49		
Most Common Abnormality in each organ				
Esophagus : Reflux esophagitis				
Stomach : H. pylori gastritis				
Duodenum: Celiac disease				
The results that affected the management (N = 56, 42.4%)				
Eosinophilic esophagitis (2)				
H.pylori gastritis (37)				
Celiac disease (17)				

*Positive predictors in multivariate analysis with *p*-value < 0.05

†Some patients might have more than one indication

According to the ESGE/ESPGHAN guidelines, 108 (13.9%) of the procedures were considered unsuitable. Appropriate indication was a sensitive indicator for abnormal endoscopic and histopathological abnormalities (85.2% and 90.1% respectively), but not specific (13.1% and 23.2% respectively) (Table 7). Serious side effects were not reported in any of the patients.

**Table 7:** Appropriate indication of endoscopic procedures as a predictor of abnormal endoscopic or pathological findings

	Abnormal endoscopic findings (n)	Normal endoscopic findings (n)
Appropriate indication	3.17	351
Inappropriate indication	55	53
Sensitivity% (95% CI)	85.2 (81.2–82.6%)	
Specificity % (95% CI)	13.1 (9.9–16.7%)	
Positive predictive value	47.8%	
Negative predictive value	47.1%	
Appropriate indication	391	183
Inappropriate indication	43	56
Sensitivity % (95% CI)	90.1% (86.9–92.7%)	
Specificity % (95% CI)	23.4% (18.2–29.3%)	
Positive predictive value	68.1%	
Negative predictive value	56.6%	

## Discussion

Pediatric endoscopy is a safe and effective diagnostic tool for children with symptomatic upper gastrointestinal lesions. Although the utility of upper endoscopy in children is on the rise, data on patients from the Middle East are scarce. This study describes the indications as well as the endoscopic and histopathological yields of upper endoscopy in patients treated at our center in Jordan. In addition, this study assesses the appropriate indication of upper endoscopic procedures according to published guidelines. [2]

Abdominal pain is one of the most common complaints of patients who present to a pediatric gastroenterology clinic. A significant portion of these children undergo upper endoscopy. In our cohort, abdominal pain is the most common indication for upper endoscopy. Sheiko et al. reported that more than one-third of children underwent endoscopy at their facility due to abdominal and epigastric pain.[5] In addition, abdominal pain is the most common indication for upper endoscopy in UK, Columbian, and US cohorts [6–8].

A previous study from northern Jordan by Rawashdeh et al. published in 1996 reported that small intestinal biopsies surpassed abdominal pain as an indication for upper endoscopy. [9] We believe the difference between our current report and what was reported by Rawashdeh et al. represents the current indication in Jordan. In addition, this may reflect the effect of the development of very sensitive and specific serological markers for celiac disease, which can improve the determination of patients who are in need of intestinal biopsies.

Endoscopic examination provides a specific morphological description of upper intestinal pathologies. Sheiko et al. [5] found 37% abnormal endoscopic findings in their cohort, with the esophagus as the most prevalent organ. Meanwhile, Lyon et al. [8] reported overall endoscopic abnormalities at 54.5%. In our cohort, less than half of the patients have abnormal endoscopy findings. Both Sheiko [5] and Lyon [8] reported an increased chance of abnormal endoscopy as the patient ages. In our population, bimodal trend was observed; children younger than 60 months and those older than 120 months were associated with a higher chance of having abnormal endoscopic findings. In our cohort the accuracy of abnormal endoscopy in detecting abnormal histopathology was around 67%. This might reflect targeted lesion biopsies taken.

Indications for endoscopy influenced the chance of having abnormal endoscopic findings. Consistent with previous reports [5, 8], abdominal pain, dysphagia and heart burn were predictive of abnormal endoscopy in multivariate analysis. In addition, we found no association between sexes or the number of indications with abnormal endoscopic findings, which is in agreement with previous reports [5, 8].

In our cohort, abdominal pain is associated with a higher chance of abnormal endoscopic findings. The diagnostic yield of upper endoscopy in abdominal pain is highly affected by patient selection. A previous study from Iran reported that 84% of patients with recurrent abdominal pain had abnormal endoscopic findings, whereas another study from Kuwait reported 68% [10]. In a study by Alabdrazak et al., abdominal pain was the most common referral symptom and the best predictor of positive upper endoscopy, reaching an accuracy level of 79.9%.[11].

Wang et al. reported a 28% rate of abnormal histological findings in children with newly presenting gastrointestinal symptoms, which is consistent with other reports from the developed world. [5, 6, 12]. However, Lyon found an overall histological abnormalities rate at 59.1% and that gastric pathologies are the most common. In our study, almost 40% of our patients have an abnormal histopathological finding, with the stomach as the most commonly affected organ.

Diagnostic concordance between endoscopic and histological findings may not be highly reliable. [13] In our study, abnormal endoscopy has a sensitivity of 67% in predicting abnormal histology. However, the endoscopic indication may affect histological findings. In our study, abdominal pain, vomiting, dysphagia, and heartburn are associated with abnormal histological findings. These findings are in agreement with Sheiko et al. [5].

The utility of endoscopic biopsies in otherwise normal endoscopic exam has not been well studied A previous study from Canadian Adult Endoscopy Unit evaluated utility of endoscopic biopsies in adult patients with normal upper endoscopy. The authors concluded that the yield is highly affected by the site of the biopsies. The added expenses mandate high selectivity to augment the yield [14]. Due to the different study population the reported predictors of abnormal histology in this study can`t be extrapolated into pediatric population.

In our study; the biopsy yield was affected by the biopsy site. The stomach represented the most common site followed by the duodenum and the esophagus. Reflux esophagitis, H.pylori gastritis and celiac disease where the most common diagnoses. In multivariate analysis of features that could predict abnormal histopathology in our children with normal endoscopic exam; younger age group and abdominal pain were positive predictors. The small size of the group and the absence of cost-effectiveness evaluation limit generalization of our results and warrant further evaluation of such group.

The classification of suitability of endoscopic procedures would help practitioners identify which patients should undergo endoscopic evaluation. In our situation, financial resources are limited and the small endoscopy team can become overwhelmed with referrals. Jantchou et al. [4] found that 13.9% of the upper endoscopies performed at their unit were inappropriate according to Paediatric Hepatology, Gastroenterology, and Nutrition Group (GFHGNP) recommendations. Inappropriate procedures were associated with a less likelihood of detecting gastrointestinal abnormalities.[4] Meanwhile, Miele et al. found that almost one-fourth of performed endoscopic procedures were inappropriate.[15] In our cohort, compared to the 2016 ESGE/ESPGHAN published guidelines [2], only 13.6% of performed procedures have been found to be inappropriate. In our cohort, appropriate indication shows good sensitivity for predicting endoscopic and histopathological abnormalities but poor specificity.

Further studies addressing the effect of upper endoscopy results on the patient management and reporting milder post-procedure complications are needed to improve the diagnostic yield and the procedure outcome. Evaluating patients referred for endoscopy at the pediatric gastroenterology clinic prior to endoscopy enlisting might decrease unnecessary procedures and costs.

Our study provides valuable data on the epidemiology, endoscopic, and pathological findings of upper endoscopy and the correlation between endoscopic and histological findings. However, the study has some weaknesses. First, this study uses a retrospective design. Second, endoscopic description is sometimes subjective and depends on the endoscopist, which may lead to the misclassification of the findings. Third, this study did not collect data about any pre-endoscopic treatments, which could have led to discordance between endoscopic and histopathological findings. Fourth, the inconsistent number and sites of endoscopic biopsies, which cannot be fully explained due to the retrospective nature of the study, limit the generalization of these study results. And lastly, this study didn`t quantify the effect of the procedure on the patients` management plan. Even normal endoscopy provides significant input by refuting a diagnosis or modifies treatment.

## Conclusions

Pediatric upper endoscopy is a valuable diagnostic tool. Older children are associated with a higher chance of abnormal endoscopic findings. Abdominal pain is the most common indication for upper endoscopy and is associated with a higher chance of abnormal endoscopy. Concordance between endoscopic and histopathological findings is not high, so routine biopsies need to be considered. Compliance with the published guidelines could improve pathology detection rates and reduce unnecessary referrals. Further studies on the effect of the endoscopy results on the management decisions are needed.

## Data Availability

The datasets generated and/or analysed during the current study are not publicly available due to the regulations of the IRB but are available from the corresponding author on reasonable request.

## Abbreviations

KAUH: King Abdullah University Hospital
JUST: Jordan University of Science and Technology
ESGE: European Society of Gastrointestinal Endoscopy
ESPGHAN: European Society for Pediatric Gastroenterology, Hepatology, and Nutrition
